# An Interplay Between Pericytes, Mesenchymal Stem Cells, and Immune Cells in the Process of Tissue Regeneration

**DOI:** 10.1155/ancp/4845416

**Published:** 2025-04-09

**Authors:** Vladislav Volarevic, Carl Randall Harrell, Aleksandar Arsenijevic, Valentin Djonov

**Affiliations:** ^1^Center for Harm Reduction of Biological and Chemical Hazards, Department of Genetics and Department of Microbiology and Immunology, Faculty of Medical Sciences, University of Kragujevac, 69 Svetozar Markovic Street, Kragujevac, Serbia; ^2^Regenerative Processing Plant, LLC 34176, US Highway 19 N, Palm Harbor, Florida, USA; ^3^Institute of Anatomy, University of Bern, Baltzerstrasse 2 3012, Bern, Switzerland

**Keywords:** exosomes, immune cells, mesenchymal stem cells, pericytes, tissue regeneration, tissue-resident stem cells

## Abstract

Immediately after injury, damaged cells elicit tissue regeneration, a healing process that enables optimal renewal and regrowth of injured tissues. Results obtained in a large number of experimental studies suggested that the cross talk between pericytes, mesenchymal stem cells (MSC), tissue-resident stem cells, and immune cells has a crucially important role in the regeneration of injured tissues. Pericytes, MSCs, and immune cells secrete bioactive factors that influence each other's behavior and function. Immune cells produce inflammatory cytokines and chemokines that influence pericytes' migration, proliferation, and transition to MSC. MSC releases immunoregulatory factors that induce the generation of immunosuppressive phenotype in inflammatory immune cells, alleviating detrimental immune responses in injured tissues. MSC also produces various growth factors that influence the differentiation of tissue-resident stem cells into specific cell lineages, enabling the successful regeneration of injured tissues. A better understanding of molecular mechanisms that regulate crosstalk between pericytes, MSC, and immune cells in injured tissues would enable the design of new therapeutic approaches in regenerative medicine. Accordingly, in this review paper, we summarized current knowledge related to the signaling pathways that are involved in the pericytes' activation, pericytes-to-MSC transition, differentiation of tissue-resident stem cells, and MSC-dependent modulation of immune cell-driven inflammation, which are crucially responsible for regeneration of injured tissues.

## 1. Introduction

Tissue injury, which occurs as a result of physical trauma, infection, inflammation, ischemia, chemical exposure, or autoimmune reactions, can lead to cellular damage, disruption of normal tissue architecture, impaired organ function, and activation of systemic and severe life-threatening inflammatory response [1]. Immediately after injury, damaged cells elicit tissue regeneration, a healing process that enables optimal renewal and regrowth of injured tissues [1, 2]. Tissue regeneration prevents scar formation and organ dysfunction, significantly improving patient outcomes and quality of life [1].

Results obtained in a large number of experimental studies suggested that the cross talk between pericytes, mesenchymal stem cells (MSC), tissue-resident stem cells, and immune cells has a crucially important role in the regeneration of injured tissues [3–7]. Upon injury, under the influence of growth and chemotactic factors, tissue-resident stem cells are mobilized from their niches toward the site of injury [6]. Stem cells secrete various bioactive factors that promote tissue healing and modulate the phenotype and function of inflammatory immune cells, which are attracted to the site of injury by damage-associated molecular patterns (DAMPs) and alarmins released from damaged parenchymal cells and endothelial cells (ECs) [3, 6, 7].

DAMPS and alarmins induce generation of inflammatory phenotype in tissue-infiltrated immune cells (macrophages, neutrophils, dendritic cells (DCs), T lymphocytes, natural killer (NK), NK T (NKT) cells), enabling massive production of vasoactive factors and inflammatory cytokines which induce changes in phenotype and function of pericytes, perivascular cells which wrap around the ECs that line the inner walls of capillaries [6]. Under the influence of inflammatory mediators, pericytes increase the synthesis of contractile proteins, detach from blood vessels, and differentiate in MSCs [4].

**Figure 1:**
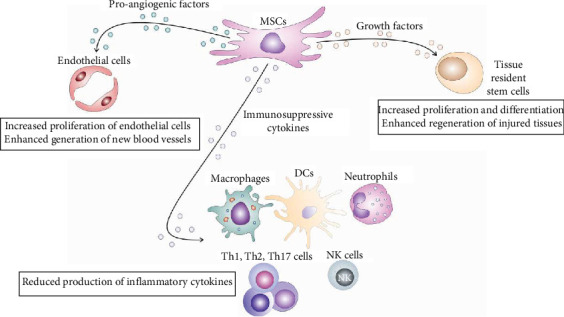
Therapeutic potential of MSCs. MSCs secrete a range of factors that have immunosuppressive, pro-angiogenic, and trophic effects. These factors work to suppress harmful immune reactions, reduce existing inflammation, promote the formation of new blood vessels, support the growth and differentiation of tissue-specific stem cells, and facilitate improved healing and regeneration of damaged tissues.

MSCs are immunoregulatory adult stem cells that produce various immunosuppressive, pro-angiogenic, and trophic factors that inhibit detrimental immune response, attenuate ongoing inflammation, induce the generation of new blood vessels and stimulate proliferation and differentiation of tissue-specific, resident stem cells, enabling enhanced repair and regeneration of injured tissues (Figure 1) [8].

Since the interaction between pericytes, MSCs, tissue-resident stem cells, and immune cells are crucially responsible for the generation of the healing process in injured tissues [3–7], a better understanding of molecular mechanisms that regulate cross-talk of these cells will enable the design of new therapeutic approaches in regenerative medicine. Accordingly, in this review paper, we summarized current knowledge related to the signaling pathways involved in (i) activation, contraction, and migration of pericytes; (ii) pericyte-to-MSC transition; and (iii) immunoregulatory, pro-angiogenic, and regenerative properties of MSCs that are crucially responsible for attenuation of immune cell-driven inflammation and enhanced regeneration of injured tissues. An extensive literature review was carried out in August 2023 across several databases (MEDLINE, EMBASE, and Google Scholar) from 1990 to the present. Keywords used in the selection were: “pericytes,” “MSCs,” “immune cells,” “tissue-resident stem cells,” “pericyte-to-MSC transition,” “tissue injury,” “blood vessel injury,” “signaling pathways,” “inflammation,” “immunosuppression,” “immunoregulation,” “neo-angiogenesis,” and “tissue repair and regeneration.” All journals were considered and an initial search retrieved 518 articles. The abstracts of all these articles were subsequently reviewed by two of the authors (Vladislav Volarevic and Carl Randall Harrell), independently to check their relevance to the subject of this manuscript. Eligible studies had to delineate molecular and cellular mechanisms that regulate the cross talk between pericytes, MSCs, tissue-resident stem cells, and immune cells in injured tissues and their findings were analyzed in this review.

## 2. Molecular Mechanisms Responsible for Changes of Pericytes' Phenotype and Function in Injured Tissues

**Figure 2:**
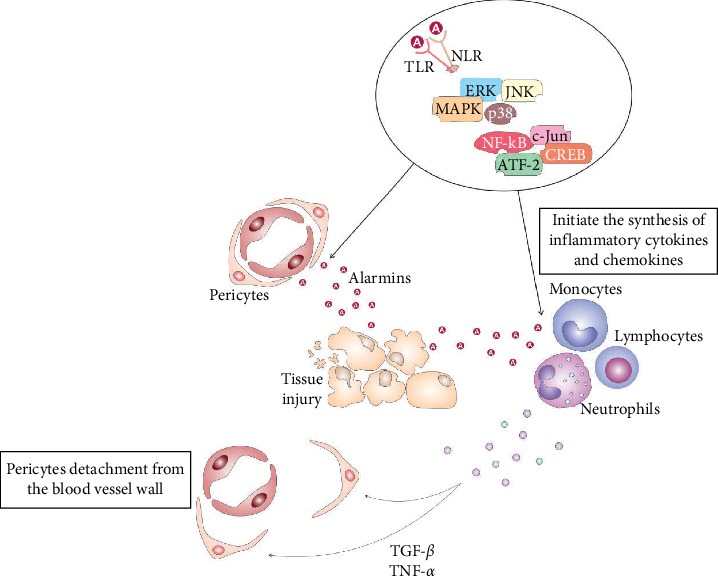
Molecular mechanisms responsible for immune cell-dependent detachment of pericytes. Alarmins, released from injured parenchymal cells, directly bind to Toll-like receptors (TLRs) and NOD-like receptors (NLRs) of pericytes and immune cells, initiating phosphorylation and consequent activation of extracellular signal-regulated kinase (ERK), c-Jun N-terminal kinase (JNK), and p38 mitogen-activated protein kinase (MAPK). Upon activation, ERK, JNK, and MAPK kinases phosphorylate NF-*κ*B, c-Jun, ATF-2, and CREB transcription factors, which initiate the synthesis of inflammatory cytokines and chemokine. Inflammatory cytokines induce conformational changes in G-protein-coupled receptors (GPCRs) and voltage-gated calcium channels in pericytes, enhancing the synthesis of myosin light chain (MLC) and myosin phosphatase target subunit 1 (MYPT1), which increase actomyosin contractility and pericyte constriction. TGF-*β* and TNF-*α* downregulate the expression of integrins on pericytes, promoting their detachment from the blood vessel wall. Chemokines (CXCL12, CXCL8, CCL2, and platelet-derived growth factor (PDGF)) act as chemoattractants that bind to their receptors on pericytes (CXCR4, CCR1, CCR2, and *PDGFR-β*), activate MAPK and ERK-driven intracellular signaling pathways that promote cytoskeletal rearrangement and cell motility.

DAMPs and alarmins, including high-mobility group box 1 (HMGB1), heat shock proteins (HSPs), S100 proteins, uric acid, and adenosine triphosphate (ATP), fragments of DNA and RNA, which are released from injured epithelial, parenchymal, and ECs, act as danger signals, alerting tissue-resident stem cells and immune cells to the tissue damage or cellular stress [9, 10]. DAMPs and alarmins directly bind to pattern recognition receptors (PRRs, including Toll-like receptors (TLRs), NOD-like receptors (NLRs), RIG-I-like receptors (RLRs), and C-type lectin receptors (CLRs)) which are expressed on the membrane of pericytes and immune cells, triggering intracellular signaling cascades, that lead to the initiation of healing process in injured tissues (Figure 2) [9, 10]. While transitioning from a quiescent state to an activated state, pericytes can exhibit increased proliferation, migration, and the production of extracellular matrix (ECM) components, potentially contributing to tissue repair or fibrosis depending on the specific injury and microenvironment. This can include functions like stabilizing damaged blood vessels, promoting angiogenesis, or participating in inflammatory responses by interacting with immune cells.

Precisely, DAMPs-dependent activation of TLRs and NLRs initiate phosphorylation and consequent activation of extracellular signal-regulated kinase (ERK), c-Jun N-terminal kinase (JNK), and p38 mitogen-activated protein kinase (MAPK) which, in turn, activate transcriptional factors nuclear factor-kappa B (NF-*κ*B), c-Jun, ATF-2, and CREB that translocate into the nucleus, bind to DNA and initiate the transcription and translation of inflammatory cytokines (tumor necrosis factor-alpha (TNF-*α*), interleukin (IL)-1*β*, IL-6, IL-18) and chemokines (C–C motif chemokine ligand (CCL)-2, CCL3, CCL5, C–X–C motif chemokine ligand (CXC)-8, CXC10) [4, 9]. Inflammatory cytokines and chemokines induce conformational changes in G-protein-coupled receptors (GPCRs) and voltage-gated calcium channels, promoting intracellular calcium release from the sarcoplasmic reticulum [9]. Additionally, TNF-*α*, IL-1*β*, and IL-18 induce activation of several signaling pathways, which enhance the synthesis of contractile proteins in pericytes [4, 9]. RhoA-ROCK (Rho-associated protein kinase) cascade activates myosin light chain (MLC) and myosin phosphatase target subunit 1 (MYPT1), which increases actomyosin contractility and pericyte constriction [4, 9]. Protein kinase C (PKC) is also activated in pericytes in response to inflammatory cytokines and DAMPs-PRRs mediated signaling. PKC phosphorylates MLC and MYPT1 and increases pericyte contractility [4, 9]. ATP-dependent activation of purinergic receptors significantly enhances calcium influx in pericytes. An increase in calcium levels triggers the activation of MLC kinase (MLCK), which phosphorylates MLC and enables interaction between actin and myosin filaments, resulting in enhanced pericyte contraction [4, 9]. Pericyte contraction leads to the narrowing of the blood vessel lumen, resulting in vasoconstriction, which reduces blood flow to the injured area, limits bleeding, and prevents excessive fluid leakage into the surrounding tissue [11]. By constricting the blood vessel, pericytes can restrict the entry of immune cells and inflammatory mediators into the injured area, thereby, modulating the inflammatory cascade and preventing excessive inflammation [11]. Also, constricted blood vessels provide a favorable environment for platelet aggregation and clot formation, promoting hemostasis and preventing further bleeding [4, 5, 11].

In addition to their effects on pericyte contraction, immune cell-derived cytokines and chemokines play a crucial role in promoting pericyte motility, detachment from blood vessels, and the pericyte-to-MSC transition [12]. Transforming growth factor beta (TGF-*β*) and TNF-*α* downregulate the expression of adhesion molecules (integrins) on pericytes, promoting their detachment from the blood vessel wall. These inflammatory mediators induce increased activity of several proteases (cathepsins, A disintegrin and metalloproteinase (ADAM), and plasmin), which degrade integrins or disrupt the interaction between integrins and their ligands, leading to the detachment of pericytes from the blood vessel wall [11]. Similarly, TNF-*α* and IL-1*β* induce increased synthesis and release of matrix metalloproteinases (MMPs) from injured ECs. MMPs degrade the ECM components that anchor pericytes to ECs, facilitating their detachment and migration away from the blood vessel [12, 13]. Chemokines (CXCL12, CXCL8, CCL2, and platelet-derived growth factor (PDGF)) act as chemoattractants that bind to their receptors on pericytes (CXCR4, CCR1, CCR2, and PDGF receptor-beta (PDGFR-*β*)), activate MAPK and ERK-driven intracellular signaling pathways that promote cytoskeletal rearrangement and cell motility [12, 13] Additionally, epigenetic modifications, such as DNA methylation and histone acetylation, also regulate the expression of integrins on pericytes. Inflammatory cytokines and chemokines may induce changes in the epigenetic landscape of pericytes, which leads to the silencing of integrin genes [12]. Downregulated expression of integrins results in the pericytes' detachment from the blood vessel walls [12, 13].

Emerging evidence suggests that, upon detachment, pericytes primed by immune cell-derived cytokines differentiate into MSCs in injured tissues [12, 14]. This differentiation process, known as pericyte-to-MSC transition or pericyte plasticity, is considered crucially important for scarless healing of injured tissues [12].

## 3. Pericyte-to-MSC Transition: The Initial Step in Tissue Regeneration

The pericyte-to-MSC transition is influenced by several cytokines and growth factors (TGF-*β*, IL-1*β*, IL-10, IL-6, vascular endothelial growth factor (VEGF), PDGF, fibroblast growth factor (FGF)-2) which play a crucial role in regulating the differentiation, proliferation, migration, and immunomodulatory properties of pericytes during their transition to MSC [12, 15].

PDGF and FGF2 are involved in various cellular processes, including cell proliferation and differentiation [16]. IL-6 and VEGF are multifunctional cytokines responsible for neo-angiogenesis in injured tissues. PDGF, FGF2, IL-6, and VEGF promote the proliferation, migration, and differentiation of pericytes, facilitating their transition into MSCs [12, 16]. FGF2 and IL-6 induce the upregulation of MSC markers (CD73 (ecto-5'-nucleotidase), CD90 (Thy-1), CD105 (endoglin), and CD44 (hyaluronan receptor)) in pericytes [12, 16]. Upregulation of these markers is associated with the pericytes' acquisition of MSC characteristics, such as multipotency and tissue repair capabilities [16]. IL-10 is an anti-inflammatory cytokine known for its immunosuppressive effects. It can influence the pericyte-to-MSC transition by promoting the acquisition of immunomodulatory properties in pericytes. IL-10 stimulates the upregulation of immunomodulatory molecules and enhances the immunosuppressive functions of MSC derived from pericytes. In addition to IL-10, pro-inflammatory cytokines (IL-1*β* and IL-6) are also able to enhance the immunomodulatory properties of pericytes during their transition to MSC, enabling MSC-dependent attenuation of ongoing inflammation in injured tissues [16].

TGF-*β*, a multifunctional cytokine involved in cell proliferation, differentiation, migration, and ECM production, is considered crucially important for pericyte-to-MSC transition in injured tissues [15, 16]. Macrophages and DCs massively produce TGF-*β* in response to injury-related signals. Inflammatory cytokines (TNF-*α* and IL-1*β*), oxidative stress, and hypoxia activate NF-kB and hypoxia-inducible factor (HIF) pathways which stimulate TGF-*β* secretion. Epigenetic modifications, such as DNA methylation and histone modifications, which occur in injured tissues, significantly increase TGF-*β* expression in tissue-resident macrophages and DCs [15, 16].

TGF-*β* is secreted in an inactive and latent form, bound to latency-associated peptides (LAPs). MMPs, plasmin, and thrombospondin-1 cleave LAPs, releasing the active form of TGF-*β*. Injured tissues undergo ECM remodeling, which induces the release of TGF-*β* from its latent form, contributing to increased TGF-*β* availability in the injured tissue [15, 17]. Once secreted, TGF-*β* may act in an autocrine and paracrine manner to further stimulate its own production and secretion. TGF-*β* binds to its receptors and activates Smad and non-Smad signaling pathways, which are both involved in pericyte-to-MSC transition [17]. In the Smad-dependent pathway, the interaction between TGF-*β* and its type II receptor (TGFBR2) activates activin receptor-like kinase 5 (ALK5), which phosphorylates and activates the type I receptor (TGFBR1)/ALK receptor complex [17]. Phosphorylated TGFBR1/ALK5 complex then phosphorylates receptor-regulated Smads (R-Smads), specifically Smad2 and Smad3. Phosphorylated R-Smads form complexes with the common mediator Smad (Co-Smad), Smad4 [17]. The Smad complex translocates into the nucleus, where, along with other transcriptional regulators, binds to specific DNA sequences (Smad-binding elements) in the promoter regions of target genes, leading to the transcriptional regulation of target genes [17]. In addition to Smad-dependent pathway, TGF-*β*/TGF-*β*Rs interactions elicit activation of non-Smad signaling, including activation of phosphatidylinositol-3 kinase (PI3K), protein kinase B (Akt), TAK1, MAPK kinases (ERK, JNK, and p38 MAPK), and Rho-like GTPases (RhoA, Rac1, and Cdc42), which are involved in cytoskeletal rearrangement, cell migration, and ECM remodeling [15, 17]. All TGF-*β*-dependent signaling pathways work together to mediate the diverse cellular responses in pericytes during their transition to MSC. While non-Smad pathways regulate pericyte interaction with neighboring cells (detachment from blood vessels, migration, interaction with ECs, and ECM), the Smad-dependent pathway primarily affects genes that regulate stemness, pluripotency, and secretory profile of newly formed MSC [15, 17].

During the pericyte-to-MSC transition, pericytes undergo significant changes in gene expression as they acquire the characteristics of MSCs [12, 15]. Common pericytes' markers, such as neuron-glial antigen 2 (NG2), PDGFR-*β*, and regulator of G protein signaling 5 (RGS5), which play a crucial role in pericyte function (attachment to blood vessel wall, proliferation, survival, angiogenesis, ECM remodeling, and cell signaling), are typically reduced in expression as pericytes shift towards the MSC [12, 15]. The changes in gene expression are responsible for altered pericytes' morphology, phenotype, and function [12, 15]. As pericytes transit into MSCs, their spindle-like and elongated shape may become more rounded or fibroblast-like [14, 15]. This change in cell shape reflects the shift from a contractile and supportive role around blood vessels to a more migratory and tissue-repairing phenotype. During the transition, pericytes may undergo an increase in cell size, becoming larger and more similar in size to MSCs. The change in cell size is associated with the acquisition of a more proliferative and multipotent phenotype [14, 15]. Pericytes undergoing the transition exhibit changes in cytoplasmic characteristics, including increased cytoplasmic volume and alterations in organelle distribution. Their cytoplasm contains more mitochondria, endoplasmic reticulum, and Golgi apparatus, reflecting the increased metabolic and secretory activities associated with the MSC phenotype [14, 15]. During the transition, pericytes exhibit increased migratory behavior due to changes in cytoskeletal organization, including the reorganization of actin filaments, reduced expression of integrins, and decreased production of ECM components (collagen (COL) and fibronectin (FN)). These changes reflect the shift in pericyte function from ECM support to a more dynamic and regenerative role [14, 15].

**Figure 3:**
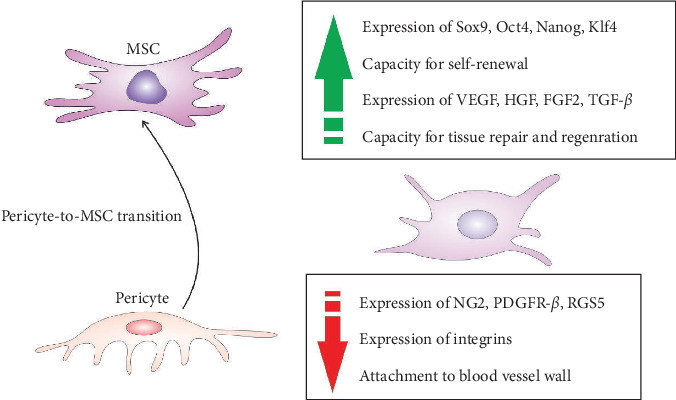
Changes in phenotype and function of pericytes during their transition to MSCs. The process of pericyte-to-MSC transition is characterized by increased expression and activity of transcriptional factors which regulate pluripotency and proliferation (Sox9, Oct4, Nanog, and Klf4), immunosuppression and angiomodulation (VEGF, HGF, FGF2, and TGF-*β*), and by attenuated expression of common pericytes' markers (NG2, platelet- PDGFR-*β*, and RGS5) and integrins which regulate pericytes' attachment to blood vessel walls.

Significantly enhanced expression of transcriptional factors (Sox9, Oct4, Nanog, and Klf4), which are associated with the stemness and pluripotency of MSC and increased expression of genes that encode growth factors capable of inducing neo-angiogenesis and differentiation of tissue-resident stem cells (VEGF, hepatocyte growth factor (HGF), FGF2, and TGF-*β*) are observed in pericytes during their transition to MSC (Figure 3) [14]. Upregulation of these factors crucially contributes to the generation of potent regenerative properties in newly formed MSCs [12, 15]. Also, during their transition to MSC, pericytes exhibit changes in the expression of genes involved in ECM remodeling [14]. This includes the upregulation of genes that are responsible for ECM degradation (MMP2 and MMP9) and ECM synthesis (COL1A1, COL3A1, and FN 1). Additionally, the pericyte-to-MSC transition involves up-regulation of genes involved in TGF-*β* (TGFBR2, SMAD2-7: ID1-3, and CDKN1A), Wnt (Wnt3A, Wnt5A, Wnt10B, AXIN2, TCF7, and FZD4), and Notch signaling (NOTCH1, NOTCH3, JAG1-2, DLL1, DLL4, HES1, HES5, and HEY1-2), which regulate proliferation and differentiation of MSCs [12, 14, 15]. Activation of the Notch pathway has been shown to upregulate the expression of the antiapoptotic BCL-2 gene, ensuring the long-term viability of MSC [14]. Activation of the Notch pathway can induce the expression of cyclins and cyclin-dependent kinases that drive cell proliferation, maintaining the self-renewal capacity of MSC [12, 14, 15]. Notch signaling can also sustain the undifferentiated state of MSCs and prevent their premature differentiation by promoting the expression of pluripotency factors, such as Oct4 and Nanog [12, 15]. While the Notch-dependent signaling cascade regulates the viability and proliferation of MSC, TGF-*β*-driven signaling controls the differentiation of MSC. TGF-*β* upregulates the expression of osteogenic (Runx2 and osteocalcin) and chondrogenic markers (Sox9 and COL II) in MSCs, promotes the synthesis of bone matrix proteins and cartilage matrix components, facilitating MSC-dependent bine mineralization and of cartilage tissue formation [12, 14, 15]. Also, TGF-*β* regulates the expression of key adipogenic transcription factors (PPAR*γ* and C/EBP*α*) in MSC and is crucially responsible for the early stages of MSC's adipogenic differentiation. Accordingly, during the transition to MSC, pericytes become multipotent cells capable of spontaneously differentiating into mesodermal cell lineages [14, 15].

Finally, during the pericyte-to-MSC transition, pericytes undergo alterations in the gene expression of cytokines, chemokines, and immunomodulatory molecules [12, 14, 15]. Upregulated expression of genes that are responsible for the synthesis of immunoregulatory molecules: (IL-10, TGF-*β*, PGE2, indoleamine-2,3-dioxygenase (IDO), programmed death-ligand 1 (PD-L1), and downregulated expression of genes that encode inflammatory cytokines (TNF-*α*, IL-1*β*, and IL-6) are observed in pericytes during their transition to MSC [14, 15]. Also, these pericytes exhibit changes in the expression of chemokines which are involved in immune cell recruitment and migration [12, 14]. There is an upregulation of CCL2, CCL5, and CXCL12, which attract monocytes, lymphocytes, and DCs to the site of tissue injury [14]. In this way, newly formed MSCs are able to directly interact with tissue-infiltrated immune cells and, in a juxtacrine and paracrine manner, modulate their inflammatory phenotype and function [14, 15].

## 4. MSC-Dependent Suppression of Inflammatory Immune Cells in Injured Tissues

MSCs are immunoregulatory stem cells with potent migratory characteristics [18]. MSC express various chemokine receptors (CXCR4, c-Met, PDGFRs, VEGFRs, CCR2, CXCR1, CXCR2, and C5aR), which enable their rapid recruitment from the periphery to the site of injury and inflammation [18]. Stromal-derived factor-1 (SDF-1/CXCL12) is a chemokine produced by injured cells. It plays a crucial role in attracting MSC [3, 18]. SDF-1 binds to its receptor, CXCR4, expressed on MSC, promoting their migration towards the site of injury. In a similar manner as SDF-1, HGF also acts as a chemoattractant for MSCs [3]. HGF binds to its receptor, c-Met, on MSC, activating signaling pathways that induce cytoskeletal rearrangement and promote migration and homing of MSCs to the injured tissue. PDGF and VEGF are growth factors released by platelets and pericytes in injured blood vessels [3]. PDGF and VEGF bind to their receptors (PDGFR-*α*, PDGFR-*β*, VEGFR1, and VEGFR2) on MSC, triggering signaling pathways that promote migration and recruitment of MSC [3, 18]. DAMPs-dependent activation of tissue-resident macrophages results in massive secretion of CCL2 and IL-8, which, in CCR2, CXCR1, and CXCR2-dependent manner, attract circulating MSC to the site of injury. In addition to these inflammatory cytokines, C5a, a protein fragment released from the cleavage of complement component C5, can attract MSCs to the site of tissue injury. C5a binds to its receptor, C5aR, on MSCs, stimulating their migration towards the injured tissue [3, 18].

MSCs are not constitutively immunosuppressive cells [8, 19]. Their phenotype and function depend on the microenvironment to which they are exposed [19]. In injured tissues, various inflammatory cytokines (TNF-*α*, IL-1*β*, IFN-*γ*, and IL-6), immunoregulatory cytokines (TGF-*β* and PGE2), hypoxia, and components of ECM (FN and COL) collectively contribute to the generation of an immunosuppressive phenotype in both pericytes-derived MSC and chemokines-recruited MSC [8, 14, 19]. These bioactive molecules induce increased synthesis of immunomodulatory molecules (IL-10, TGF-*β*, IDO, and PGE2), enabling MSC to suppress detrimental immune responses and promote tissue regeneration [8, 19].

MSC-derived IL-10, TGF-*β*, and PGE2 suppress the production of pro-inflammatory cytokines (TNF-*α*, IL-1*β*, and IL-12) in macrophages and promote their transition to alternatively activated, anti-inflammatory, and tissue-regenerative phenotype [19, 20]. MSC-primed, alternatively activated (M2) macrophages produce IL-10, TGF-*β*, IL-4, IL-13, VEGF, FGF-2, FGF-7, and PDGF, which contribute to the resolution of inflammation and the restoration of tissue homeostasis. TGF-*β*, PDGF, FGF-2, and FGF-7 promote the production of ECM components and stimulate the differentiation of fibroblasts into myofibroblasts, which are involved in wound healing and tissue contraction [19, 20]. VEGF promotes the formation of new blood vessels, facilitating the delivery of oxygen and nutrients to the injured tissue. IL-13 promotes tissue repair by stimulating COL synthesis, promoting ECM remodeling, and enhancing angiogenesis, while IL-10, IL-4, and TGF-*β* act as anti-inflammatory cytokines that suppress the generation of inflammatory phenotype in neutrophils, DCs, and T lymphocytes [19, 20].

MSC-sourced IL-10 and TGF-*β* are mainly responsible for MSC-dependent induction of immunosuppressive and tolerogenic phenotype in DCs in injured tissues [20]. IL-10 binds to its receptor on the surface of DCs, leading to the activation of the JAK-STAT signaling pathway. This results in the phosphorylation and activation of STAT3, which in turn regulates the expression of genes involved in the generation of tolerogenic DCs [20]. These genes include SOCS3, which acts as a negative regulator of pro-inflammatory cytokine signaling, and IL-10 itself, which further enhances the tolerogenic phenotype of DCs [20]. TGF-*β* binds to its receptor on the surface of DCs, leading to the activation of the Smad signaling pathway. This results in the phosphorylation and activation of Smad proteins, which translocate to the nucleus and regulate the expression of FoxP3 and IDO genes which are crucially important for DC-dependent expansion of Tregs [20].

MSC-derived IDO depletes tryptophan in the injured tissues [20, 21]. The reduced availability of tryptophan affects the metabolic balance and alters the proliferation of inflammatory T lymphocytes, NK, and NKT cells [20, 21]. Additionally, IDO-dependent reduction of L-tryptophan inhibits the mechanistic target of the rapamycin (mTOR) signaling pathway, which reduces the activation of NLRP3 inflammasome in inflammatory macrophages and neutrophils [21]. Additionally, metabolites generated from tryptophan metabolism, such as kynurenine and quinolinic acid, suppress the production of reactive oxygen species in inflammatory immune cells. Also, kynurenine and its downstream metabolite 3-hydroxy anthranilic acid activate the aryl hydrocarbon receptor (AhR), which, in turn, attenuates the production of IL-1*β* and IL-18 in DCs, macrophages, and neutrophils [21]. Finally, MSC-sourced IDO enhances the generation and expansion of immunosuppressive Tregs since kynurenine promotes the expression of Treg lineage-defining transcription factor FoxP3 in naïve CD4+ T cells [21, 22]. Upon activation of TCRs, intracellular signaling pathways driven by protein kinase B and mTOR destabilize the immunoregulatory phenotype of resting Tregs and cause their reprogramming into a pro-inflammatory helper-like phenotype (ex-Tregs), characterized by increased production of Th1 and Th17-related inflammatory cytokines, IFN-*γ* and IL-17 [22]. By inducing low levels of tryptophan in the local microenvironment, MSC-derived IDO and kynurenine activate stress-response-induced general control nonderepressible 2 (GCN2) kinase, which suppresses protein kinase B/mTOR signaling in Tregs and prevents trans-differentiation of Tregs in inflammatory Th1 and Th17 cells [21, 22]. Additionally, since GCN2 kinase downregulates expression of TCR zeta chain in CD8+ cytotoxic T cells (CTLs), by increasing the activity of GCN2 kinase in CTLs, MSC-sourced IDO and kynurenine attenuate cytotoxic properties of CTLs and suppress CTL-dependent tissue damage [21]. In addition to IDO and IL-10, tolerogenic DCs secrete PGE2, TGF-*β*, and IL-4, which promote the generation of immunosuppressive, alternatively activated M2 macrophages and contribute to the generation of the immunosuppressive microenvironment in injured and inflamed tissues [20].

## 5. The Cross Talk Between MSC and Tissue-Resident Stem Cells in Injured Tissues

**Figure 4:**
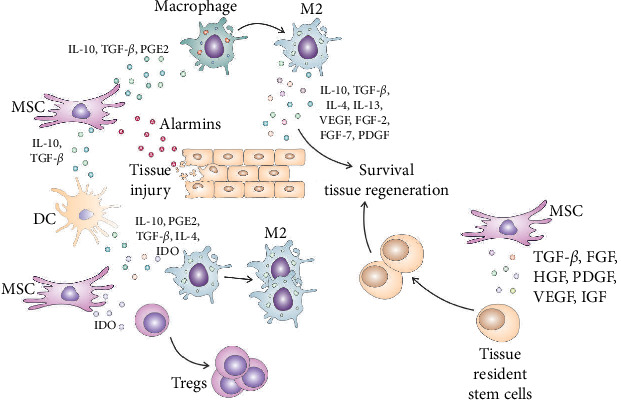
MSC-dependent modulation of tissue-resident stem cells and immune cells. Upon tissue injury, alarmins, released from injured cells, induce the generation of immunosuppressive phenotype in MSCs. MSC-derived IL-10, TGF-*β*, and PGE2 suppress the production of pro-inflammatory cytokines (TNF-*α*, IL-1*β*, and IL-12) in macrophages and promote their transition to alternatively activated, anti-inflammatory, and tissue-regenerative phenotype. MSC-primed, alternatively activated (M2) macrophages produce IL-10, TGF-*β*, IL-4, IL-13, VEGF, FGF-2, FGF-7, and PDGF, which contribute to the resolution of inflammation and the restoration of tissue homeostasis. MSC-sourced IL-10 and TGF-*β* are mainly responsible for MSC-dependent induction of immunosuppressive, tolerogenic phenotype in DCs in injured tissues. Tolerogenic DCs secrete immunosuppressive cytokines (IL-10, PGE2, TGF-*β*, IL-4, and IDO), which promote the generation and expansion of immunosuppressive T regulatory cells (Tregs) and alternatively activated macrophages. Also, MSC, in an IDO-dependent manner, induces the generation and expansion of Tregs, importantly contributing to the creation of an immunosuppressive microenvironment. Additionally, MSCs secrete a variety of growth factors (TGF-*β*, FGF, HGF, PDGF, VEGF, and insulin-like growth factor (IGF)), which synergistically act to promote the proliferation, migration, and differentiation of tissue-resident stem cells, enabling enhanced regeneration of injured tissues.

The interaction between MSC and tissue-resident stem cells in injured tissues is a complex and dynamic process that plays a crucial role in tissue regeneration (Figure 4) [23]. It involves a combination of paracrine signaling, growth factor/cytokine cross talk, direct cell-to-cell contact, and ECM remodeling [23–25]. These interactions create a microenvironment that supports the activation, proliferation, migration, and differentiation of tissue-resident stem cells, facilitating efficient tissue regeneration [25].

MSC secrete a variety of growth factors (TGF-*β*, FGF, HGF, PDGF, VEGF, and insulin-like growth factor (IGF), which synergistically act to promote the proliferation, migration, and differentiation of tissue-resident stem cells, enhancing their regenerative capacity (Table 1) [25]. It is important to note that the biological effects of MSC-derived growth factors on the survival, proliferation, and differentiation of stem cells can vary depending on the specific tissue microenvironment and the extent of an injury [25].

MSC-derived TGF-*β* is mainly responsible for MSC-dependent suppression of activated T lymphocytes and NKT cells in all injured tissues since it causes G1 cell cycle arrest of proliferated lymphocytes [20]. Within the central nervous system, MSC-sourced TGF-*β* activates neural stem cells (NSCs), regulates neurogenesis, and promotes neuronal survival [17]. The TGF-*β* signaling pathway is initiated in NSC when TGF-*β* ligands bind to TGF type II receptors into active kinases that can phosphorylate and activate type I receptors [17]. Activated type I receptors then phosphorylate receptor-regulated Smad proteins (R-Smads), leading to the formation of a complex with co-Smad4 [17]. This Smad complex then translocates to the nucleus where it regulates the transcription of target genes involved in NSC function. In addition to Smad-dependent signaling, TGF-*β* can also activate non-Smad signaling pathways such as the MAPK pathway and phosphatidylinositol-3 kinase (PI3K)/Akt pathway [17]. These pathways can modulate the activity of transcription factors and cofactors that regulate the expression of genes critical for NSC proliferation and differentiation [20]. TGF-*β* helps to maintain the population of NSC by promoting their survival and proliferation and by preventing premature differentiation [17, 20]. It can influence the fate of NSCs by promoting neuronal differentiation, inhibiting glial differentiation, or inducing a switch between neuronal and glial differentiation pathways [17, 20]. TGF-*β* is also important for cardiac regeneration since it activates the Smad-dependent pathway in cardiac stem cells (CSCs) and cardiac progenitor cells, promoting their activation, survival, and proliferation [26]. Additionally, it induces the hypertrophic growth of cardiomyocytes, leading to the enlargement of individual cardiac cells [26]. MSC, in a TGF-*β*-dependent manner, interacts with endothelial progenitor stem cells (EPCs) to promote their proliferation and differentiation, crucially contributing to the generation of new blood vessels in injured tissues [17]. Within the bone marrow, MSC-derived TGF-*β* plays a role in maintaining the hematopoietic stem cell (HSC) niche and regulating immune cell differentiation [17, 20]. TGF-*β* can inhibit the expansion of HSC by inducing cell cycle arrest in the G1 phase [17]. Additionally, TGF-*β* induces differentiation of HSC into myeloid cells and influences the migration of HSC in injured tissues by regulating the expression of adhesion molecules and chemokine receptors on their membranes [17, 20].

MSC-sourced FGF binds to FGF receptors (FGFRs) on tissue-resident stem cells, affecting their phenotype and function [20]. Upon FGF binding, FGFRs undergo conformational changes that lead to receptor dimerization [27]. This dimerization brings the intracellular kinase domains of the receptors into close proximity, allowing them to phosphorylate each other on specific tyrosine residues [27]. This autophosphorylation event further activates the kinase activity of the FGFRs. The activated FGFRs recruit adaptor protein, FGFR substrate 2 (FRS2), enabling activation of Grb2-Sos complex, which leads to the activation of the Ras-Raf-MEK cascade [27]. This cascade activates ERK kinase, which translocates to the nucleus and phosphorylates various transcription factors, modulating gene expression and promoting cellular proliferation and differentiation of tissue-resident stem cells. FGF–FGFRs complex can also activate PI3K kinase, leading to the production of phosphatidylinositol (3,4,5)-trisphosphate (PIP3) [27]. PIP3 recruits and activates Akt, a serine/threonine kinase that regulates various cellular processes in tissue-resident stem cells, including cell survival, metabolism, and migration. FGFR activation can stimulate the phosphorylation of specific tyrosine residues on the receptor, which serve as docking sites for members of the STAT family of transcription factors [27]. Phosphorylated STAT proteins translocate to the nucleus, where they regulate gene expression and mediate cellular responses such as proliferation and differentiation of stem and progenitor cells [27]. Accordingly, FGF released by MSCs induces the proliferation and differentiation of tissue-resident stem cells into specific cell lineages necessary for tissue regeneration [25]. Also, MSC-sourced FGF can act as chemoattractants, guiding the migration of tissue-resident stem cells toward the area of tissue damage [25]. It also stimulates the synthesis and deposition of ECM components (COL and FN), creating a favorable microenvironment for engraftment of stem cells by providing structural support and guidance cues for their migration, proliferation, and differentiation [25].

HGF, also known as scatter factor, is a paracrine factor secreted by MSCs that can modulate the survival, proliferation, migration, and differentiation of tissue-resident stem cells during tissue healing [20, 25]. MSC-derived HGF has antiapoptotic effects, promoting the survival of stem cells and protecting them from cell death induced by injury-related factors [25]. Upon binding to a c-Met receptor on stem cells, MSC-sourced HGF triggers the activation of the PI3K/Akt pathway [31]. Akt phosphorylates and inactivates pro-apoptotic proteins (Bad and Bax), preventing them from triggering the apoptotic cascade [31]. Additionally, HGF can upregulate the expression of antiapoptotic Bcl-2 family members, promoting cell survival by preventing the release of cytochrome c from mitochondria. MSC-derived HGF upregulates the expression of VEGF and IGF-1, which have antiapoptotic effects [25]. These induced factors further contribute to the protection of stem cells from apoptosis [25]. Also, HGF enhances the expression of integrins, which mediate cell-ECM interactions [25]. By promoting cell adhesion and providing survival signals, MSC-sourced HGF protects stem cells from apoptotic cell death [25]. HGF-mediated antiapoptotic effects enable tissue-resident stem cells to resist injury-related factors and successfully carry out their regenerative functions [25]. In addition to its antiapoptotic effects, MSC-derived HGF promotes the activation and proliferation of tissue-resident stem cells by activating the MAPK/ERK pathway [31]. Finally, MSC-sourced HGF may act as a chemoattractant since it stimulates cytoskeletal rearrangement in stem cells, enabling their migration to the site of injury [25]. HGF modulates multiple signaling pathways that converge on the regulation of actin dynamics, microtubule formation, and cell motility [25]. HGF regulates the expression and activity of Rho GTPases, which control actin polymerization, stress fiber formation, and cell adhesion [20, 25]. Additionally, HGF triggers an activation of PI3K and MAPK-dependent signaling cascades that results in the reorganization of the actin filaments and microtubules, leading to changes in stem cell shape and motility. In this way, MSC-sourced HGF coordinates the stem cells' responses to injury and drives their migration, proliferation, and differentiation [20, 25].

MSC-derived VEGF and PDGF stimulate the proliferation and migration of EPC and ECs, leading to the formation of a functional vascular network in the injured tissue [28]. Both VEGF and PDGF signaling pathways in EPCs converge on common downstream targets, such as Akt and ERK, which regulate cell survival, proliferation, and migration [29]. VEGF binds to its specific VEGF2 receptor, while PDGF binds to PDGFR*α* and PDGFR-*β*, leading to receptor dimerization and activation [29]. Activated VEGFR2 and PDGFRs recruit and activate PI3K, which generates PIP3. PIP3, in turn, activates Akt, a serine/threonine kinase that promotes cell survival, proliferation, and migration in EPCs [29]. VEGF and PDGF also activate Ras/Raf/MEK/ERK-signaling cascade in EPCs and ECs, resulting in the phosphorylation and activation of NF-kB transcription factor that regulates ECs' proliferation, survival, and angiogenesis [29]. VEGF and PDGF-driven neo-angiogenesis is crucial for supplying oxygen and nutrients to EPCs promoting their survival and regenerative functions [28, 29].

In injured tissues, MSC-derived IGF promotes the differentiation of tissue-resident stem cells by activating intracellular signaling pathways, modulating gene expression, interacting with other growth factors and cytokines, regulating the cell cycle and proliferation, and modulating epigenetic modifications [23, 30]. In a similar manner as VEGF and PDGF, MSC-derived IGF activates the PI3K/Akt signaling pathway, enhancing the proliferation of stem cells [32]. It promotes the transition of stem cells from a quiescent state (G0 phase) to an active proliferative state (G1 phase) [32]. Additionally, MSC-sourced IGF activates Smad proteins which bind to target gene promoters and initiate gene transcription [32]. The activated genes can encode proteins that drive stem cells towards specific lineages, promoting their differentiation [30]. Also, MSC-derived IGF can influence the activity of enzymes involved in epigenetic modifications, leading to changes in chromatin structure and accessibility [30]. This modulation of epigenetic marks can promote or suppress the expression of specific genes associated with stem cell differentiation [30]. For this purpose, MSC-derived IGF interacts with other growth factors (bone morphogenetic proteins (BMPs), FGFs, and TGF-*β*) which are present in the microenvironment of injured tissues [23–25]. These growth factors collectively contribute to the commitment and differentiation of resident stem cells into specific lineages, enabling successful tissue regeneration [23–25].

## 6. Conclusions and Future Perspectives

The cross talk between pericytes, MSCs, and immune cells is essential for the successful regeneration of injured tissues. This interaction is mainly driven by paracrine signaling. Pericytes, MSCs, and immune cells secrete bioactive factors that influence each other's behavior and function. Immune cells produce inflammatory cytokines and chemokines that influence pericytes' migration, proliferation, and transition to MSC. Pericyte-derived MSC releases immunoregulatory factors, which induce the generation of immunosuppressive phenotype in inflammatory immune cells, alleviating detrimental immune responses in injured tissues. MSC also produces various growth factors which influence the survival, proliferation, and differentiation of tissue-resident stem cells into specific cell lineages, enabling the successful regeneration of injured tissues.

Although significant progress has been made in understanding the molecular mechanisms that regulate cross talk between pericytes, MSCs, and immune cells in tissue regeneration, it should be noted that several important aspects are still unknown. Translation of experimental findings into clinical applications is still in the early stages. Therefore, new animal studies should be conducted in order to uncover juxtacrine interactions between these cells and to determine temporal dynamics and microenvironmental influences on this crosstalk. Understanding and harnessing the crosstalk between pericytes, MSCs, and immune cells in injured tissues could provide valuable insights for developing new therapeutic strategies that would enhance tissue regeneration and improve outcomes in regenerative medicine.

In summing up, it can be concluded that (i) inflammation-induced activation of pericytes, (ii) their differentiation in immunosuppressive MSCs, (iii) MSC-dependent suppression of inflammatory immune cells, and (iv) MSC-driven proliferation and differentiation of tissue-resident stem cells are the main cellular events crucially responsible for optimal healing of injured tissues. In the end, it should be emphasized that the main limitation of this review article is that all conclusions were drawn based on the interpretation of the results of published studies without access to individual, originally generated data, limiting the possibility of more advanced analyses of experimentally obtained findings. Also, an additional limitation of this review article is the exclusion of publications that were not written in the English language, which could result in the omission of some topic-related findings.

## Figures and Tables

**Table 1:** Effects of MSC-derived factors on tissue-resident stem cells and immune cells.

MSC-derived factor	Target cells	Mechanism of action	Biological effects	Ref. No.
TGF-*β*	T lymphocytes; NKT cells	G1 cell cycle arrest	Attenuated immune cell-driven inflammation	[17]
TGF-*β*	Neural stem cell	Activation of Smad and non-Smad/MAPK/PI3K-driven-signaling pathways	Improved survival of neural stem cells	[17, 20]
TGF-*β*	Cardiac stem cells	Activation of smad-dependent pathway	Hypertrophic growth of cardiomyocytes	[26]
TGF-*β*	Endothelial progenitor cells	Activation of smad-dependent pathway	Generation of new blood vessels	[20]
TGF-*β*	Hematopoietic stem cells	G1 cell cycle arrest	Maintaining homeostasis of bone marrow	[17]
FGF	Tissue-resident stem cells	Activation of PI3K and Ras-Raf-MEK cascades	Increased survival, and migration of stem cells	[20, 27]
HGF	Tissue-resident stem cells	Activation of Bcl-2-driven antiapoptotic pathways; activation of PI3K/Akt, MAPK/ERK pathways	Improved survival of stem cells	[20, 25]
VEGF	Endothelial progenitor cells	Activation of VEGFR2/PI3K-driven signaling	Generation of new blood vessels	[28, 29]
PDGF	Endothelial progenitor cells	Activation of PDGFR/Akt-driven signaling	Enhanced neoangiogenesis	[28, 29]
IGF	Tissue-resident stem cells	Activation of PI3K/Akt signaling pathways	Increased proliferation of stem cells; enhanced regeneration of injured tissues	[23, 30]

## Data Availability

Results discussed in this paper are available in cited articles.

## References

[1] Zhou S., Xie M., Su J., Cai B., Li J., Zhang K. (2023). New Insights Into Balancing Wound Healing and Scarless Skin Repair. *Journal of Tissue Engineering*.

[2] Hora S., Wuestefeld T. (2023). Liver Injury and Regeneration: Current Understanding, New Approaches, and Future Perspectives. *Cells*.

[3] Harrell C. R., Djonov V., Volarevic V. (2021). The Cross-Talk Between Mesenchymal Stem Cells and Immune Cells in Tissue Repair and Regeneration. *International Journal of Molecular Sciences*.

[4] Nakamura K., Ago T. (2023). Pericyte-Mediated Molecular Mechanisms Underlying Tissue Repair and Functional Recovery After Ischemic Stroke. *Journal of Atherosclerosis and Thrombosis*.

[5] Ruan Q., Tan S., Guo L., Ma D., Wen J. (2023). Prevascularization Techniques for Dental Pulp Regeneration: Potential Cell Sources, Intercellular Communication and Construction Strategies. *Frontiers in Bioengineering and Biotechnology*.

[6] Horner E., Lord J. M., Hazeldine J. (2023). The Immune Suppressive Properties of Damage Associated Molecular Patterns in the Setting of Sterile Traumatic Injury. *Frontiers in Immunology*.

[7] Zhang W., Ling Y., Sun Y., Xiao F., Wang L. (2023). Extracellular Vesicles Derived from Mesenchymal Stem Cells Promote Wound Healing and Skin Regeneration by Modulating Multiple Cellular Changes: A Brief Review.. *Genes (Basel)*.

[8] Harrell C. R., Jovicic N., Djonov V., Arsenijevic N., Volarevic V. (2019). Mesenchymal Stem Cell-Derived Exosomes and Other Extracellular Vesicles as New Remedies in the Therapy of Inflammatory Diseases. *Cells*.

[9] Land W. G. (2023). Role of DAMPs and Cell Death in Autoimmune Diseases: The Example of Multiple Sclerosis. *Genes and Immunity*.

[10] Sakai S., Shichita T. (2023). Role of Alarmins in Poststroke Inflammation and Neuronal Repair. *Seminars in Immunopathology*.

[11] Immanuel J., Yun S. (2023). Vascular Inflammatory Diseases and Endothelial Phenotypes. *Cells*.

[12] Marson R. F., Regner A. P., da Silva Meirelles L. (2023). Mesenchymal “Stem” Cells, or Facilitators for the Development of Regenerative Macrophages? Pericytes at the Interface of Wound Healing. *Frontiers in Cell and Developmental Biology*.

[13] Benabid A., Peduto L. (2020). Mesenchymal Perivascular Cells in Immunity and Disease. *Current Opinion in Immunology*.

[14] de Souza L. E., Malta T. M., Kashima Haddad S., Covas D. T. (2016). Mesenchymal Stem Cells and Pericytes: To What Extent Are They Related?. *Stem Cells and Development*.

[15] Craig D. J., James A. W., Wang Y., Tavian M., Crisan M., Péault B. M. (2022). Blood Vessel Resident Human Stem Cells in Health and Disease. *Stem Cells Translational Medicine*.

[16] Kemp S. S., Lin P. K., Sun Z. (2022). Molecular Basis for Pericyte-Induced Capillary Tube Network Assembly and Maturation. *Frontiers in Cell and Developmental Biology*.

[17] Kandasamy M., Anusuyadevi M., Aigner K. M. (2020). TGF-*β* Signaling: A Therapeutic Target to Reinstate Regenerative Plasticity in Vascular Dementia?. *Aging and Disease*.

[18] Song N., Scholtemeijer M., Shah K. (2020). Mesenchymal Stem Cell Immunomodulation: Mechanisms and Therapeutic Potential. *Trends in Pharmacological Sciences*.

[19] Gazdic M., Volarevic V., Arsenijevic N., Stojkovic M. (2015). Mesenchymal Stem Cells: A Friend or Foe in Immune-Mediated Diseases. *Stem Cell Reviews and Reports*.

[20] Volarevic V., Gazdic M., Simovic Markovic B., Jovicic N., Djonov V., Arsenijevic N. (2017). Mesenchymal Stem Cell-Derived Factors: Immuno-Modulatory Effects and Therapeutic Potential. *Biofactors*.

[21] Acovic A., Gazdic M., Jovicic N. (2018). Role of Indoleamine 2,3-Dioxygenase in Pathology of the Gastrointestinal Tract. *Therapeutic Advances in Gastroenterology*.

[22] Acovic A., Simovic Markovic B., Gazdic M. (2018). Indoleamine 2,3-Dioxygenase-Dependent Expansion of T-Regulatory Cells Maintains Mucosal Healing in Ulcerative Colitis. *Therapeutic Advances in Gastroenterology*.

[23] Maldonado P., Smith N. H., Barnes E. E. (2023). Clinical Utility of Mesenchymal Stem/Stromal Cells in Regenerative Medicine and Cellular Therapy. *Journal of Biological Engineering*.

[24] Xue Z., Liao Y., Li Y. (2024). Effects of Microenvironment and Biological Behavior on the Paracrine Function of Stem Cells. *Genes and Diseases*.

[25] Chouaib B., Haack-Sørensen M., Chaubron F., Cuisinier F., Collart-Dutilleul P. Y. (2023). Towards the Standardization of Mesenchymal Stem Cell Secretome-Derived Product Manufacturing for Tissue Regeneration. *International Journal of Molecular Sciences*.

[26] Ren L. L., Li X. J., Duan T. T. (2023). Transforming Growth Factor-*β* Signaling: From Tissue Fibrosis to Therapeutic Opportunities. *Chemico-Biological Interactions*.

[27] Mossahebi-Mohammadi M., Quan M., Zhang J. S., Li X. (2020). FGF Signaling Pathway: A Key Regulator of Stem Cell Pluripotency. *Frontiers in Cell and Developmental Biology*.

[28] Ball S. G., Shuttleworth C. A., Kielty C. M. (2007). Mesenchymal Stem Cells and Neovascularization: Role of Platelet-Derived Growth Factor Receptors. *Journal of Cellular and Molecular Medicine*.

[29] Popescu A. M., Alexandru O., Brindusa C. (2015). Targeting the VEGF and PDGF Signaling Pathway in Glioblastoma Treatment. *International Journal of Clinical and Experimental Pathology*.

[30] Hussein N. M. S., Meade J. L., Pandit H., Jones E., El-Gendy R. (2021). The Effect of Diabetes Mellitus on IGF Axis and Stem Cell Mediated Regeneration of the Periodontium. *Bioengineering (Basel)*.

[31] Cao Z., Xie Y., Yu L., Li Y., Wang Y. (2020). Hepatocyte Growth Factor (HGF) and Stem Cell Factor (SCF) Maintained the Stemness Of Human Bone Marrow Mesenchymal Stem Cells (hBMSCs) During Long-Term Expansion By Preserving Mitochondrial Function Via the PI3K/AKT, ERK1/2, and STAT3 Signaling Pathways. *Stem Cell Research & Therapy*.

[32] Hakuno F., Takahashi S. I. (2018). IGF1 Receptor Signaling Pathways. *Journal of Molecular Endocrinology*.

